# Spatial and temporal clusters of avian influenza a (H7N9) virus in humans across five epidemics in mainland China: an epidemiological study of laboratory-confirmed cases

**DOI:** 10.1186/s12879-020-05345-4

**Published:** 2020-08-26

**Authors:** Xuzheng Shan, Yongqin Wang, Ruihong Song, Wen Wei, Hongxiu Liao, Huang Huang, Chunqiong Xu, Lvlin Chen, Shiyun Li

**Affiliations:** 1 Prevention and Health Section, Affiliated Hospital, Chengdu University, Chengdu, Sichuan China; 2 Department of Epidemiology and Biostatistics, West China School of Public Health and West China Fourth Hospital, Sichuan University, Chengdu, Sichuan China; 3 Transaction Management and Information Department, Panzhihua City Center for Disease Control and Prevention, Panzhihua, Sichuan China

**Keywords:** H7N9, Cluster, Spatial, Temporal, Mainland China

## Abstract

**Background:**

Avian influenza A (H7N9) virus was first reported in mainland China in 2013, and alarming in 2016–17 due to the surge across a wide geographic area. Our study aimed to identify and explore the spatial and temporal variation across five epidemics to reinforce the epidemic prevention and control.

**Methods:**

We collected spatial and temporal information about all laboratory-confirmed human cases of A (H7N9) virus infection reported in mainland China covering 2013–17 from the open source. The autocorrelation analysis and intensity of cases were used to analyse the spatial cluster while circular distribution method was used to analyse the temporal cluster.

**Results:**

Across the five epidemics, a total of 1553 laboratory-confirmed human cases with A (H7N9) virus were reported in mainland China. The global Moran’s *I* index values of five epidemic were 0.610, 0.132, 0.308, 0.306, 0.336 respectively, among which the differences were statistically significant. The highest intensity was present in the Yangtze River Delta region and the Pearl River Delta region, and the range enlarged from the east of China to inner provinces and even the west of China across the five epidemics. The temporal clusters of the five epidemics were statistically significant, and the peak period was from the end of January to April with the first and the fifth epidemic later than the mean peak period.

**Conclusions:**

Spatial and temporal clusters of avian influenza A (H7N9) virus in humans are obvious, moreover the regions existing clusters may enlarge across the five epidemics. Yangtze River Delta region and the Pearl River Delta region have the spatial cluster and the peak period is from January to April. The government should facilitate the tangible improvement for the epidemic preparedness according to the characteristics of spatial and temporal clusters of patients with avian influenza A (H7N9) virus.

## Background

From 2013 up to now, the avian influenza A (H7N9) virus infections in humans in mainland China are unprecedented both in terms of mortality and morbidity [1–3], with the risk of continuous emerge and spread for the virological and molecular characteristics of the virus [4–8]. The A (H7N9) virus has the highest risk score among the 12 novel influence A virus assessed by the Influenza Risk Assessment Tool [9]. The risk assessment of A H7N9 is crucial for pandemic preparedness of public health, especially in the neglected locations [10].

Many researches have descripted the characteristics of the epidemics. Clinical features of human infections in China reported during the five epidemics were similar [1], and the spatial and temporal characteristics were not [11]. According to the research of the fourth epidemic, the area which had never been reported with cases before announced confirmed cases during the fourth epidemic [8]. At the same time, the start time differed across the epidemics, shifting gradually from February–April to December–March [7].

Surprisingly, although five epidemics have occurred in China, the evidence of spatial and temporal features have been gained through description methods without statistical inference [12]. Moreover, the inherent bias may be present due to the cases centralized at the prefecture or province level. Continuous epidemiological investigations should be crucial to outline the epidemic trajectory and inform public health for pandemic preparedness [13–16]. In comparison of the epidemic description on the province level, the present study aimed to assess spatial and temporal variation across five epidemics through appropriate statistical inference methods. The result can help the public understand epidemic regulations better and enhance the emergency capacity of public health in China.

## Methods

### Data sources

The data of confirmed A (H7N9) human cases across the five epidemics in mainland China were drawn from the EMPRES Global Animal Disease Information System (EMPRES-i) of the Food and Agricultural Organization (FAO) (empres-i.fao.org/eipws3g/bioclimatic). The cases only included Low pathogenic avian influenza (LPAI) H7N9. The first epidemic was defined as being from Jan 1, 2013, to Sept 30, 2013. And subsequent epidemics were defined from Oct 1 to Sept 30 of the following year. All data was drawn from publicly available data sources, supplied and analysed in an anonymous format, without access to personal identifying information. Therefore, our study was exempt from institutional review board assessment.

### Data analysis

#### Overview

To explore the variance of spatial and temporal clusters of avian influenza A (H7N9) infections in humans, we used global Moran’s *I* statistic to evaluate the spatial clusters, and circular distribution method to evaluate the temporal clusters. All statistical computations were performed using R software version 3.4.3 (R Foundation for Statistical Computing, Vienna, Austria). All codes used in the analysis are available on request from the corresponding author.

#### Spatial cluster analysis

We used spatial autocorrelation analysis and intensity of cases to analyse the spatial clusters. Global Moran’s *I* statistic is usually used in spatial autocorrelation analysis [17]. The value of *I* is between − 1 and 1. *I* > 0 is positive spatial correlation and means aggregation distribution; *I* = 0 means random distribution; *I* < 0 is negative spatial correlation and means discrete distribution.

Intensity is the average density of points (expected number of points per unit area) [18]. In general, the intensity of point will vary from place to place, so the intensity may be inhomogeneous. The kernel smoothing intensity function can be estimated nonparametrically in this study and the unit area represented per Km^2^.

#### Temporal cluster analysis

Seasonal analysis is an important part of epidemiology. Circular distribution method is a statistical method that transforms the data with periodic changes into linear data through the transformation of trigonometric function [19]. It can evaluate whether there is temporal cluster and even provide the precise kurtosis time. The central tendency is expressed by the mean angle, while the discrete tendency is expressed by the standard deviation. Table 1 expressed the conversion.

**Table 1:** Month conversion of circular distribution method

Month	Days of each month (days)	Mid-point days (days)	Median angle (degree)	Median angle (radian)
1	1–31	15.5	15.288	0.267
2	32–59	45.0	44.384	0.775
3	60–90	74.5	73.479	1.283
4	91–120	105.0	103.562	1.808
5	121–151	135.5	133.644	2.333
6	152–181	166.0	163.726	2.857
7	182–212	196.5	193.808	3.383
8	213–243	227.5	224.383	3.916
9	244–273	258.0	254.465	4.441
10	274–304	288.5	284.548	4.966
11	305–334	319.0	314.630	5.491
12	335–365	349.5	344.712	6.016

Note: The days of a year are distributed to a circular equally

## Results

### Overall incidence

Over the study period in the mainland China, a total of 1553 laboratory-confirmed human infection with A (H7N9) virus had occurred, including 134 (8.63%) cases reported in the first epidemic, 302 (19.46%) cases in second epidemic, 221 (14.43%) cases in the third epidemic, 117 (7.67%) cases in the fourth epidemic and 776 (50%) cases in the fifth epidemic. The epidemics spread to 27 provinces in mainland China. More than 60% of cases located in eastern and southern China, i.e. Zhejiang (20.9%), Guangdong (16.6%), and Jiangsu (15.4%). The detail results were present in Table 2.

**Table 2:** Five epidemics of avian influenza A (H7N9) virus in humans in 2013–17 (%)

Province	First epidemic (2013.1–2013.9)	Second epidemic (2013.10–2014.9)	Third epidemic (2014.10–2015.9)	Fourth epidemic (2015.10–2016.9)	Fifth epidemic (2016.10–2017.9)	Total	Rank
Zhejiang	45 (13.89)	93 (28.70)	46 (14.20)	36 (11.11)	104 (32.10)	324	1
Guangdong	1 (0.39)	105 (40.70)	73 (28.68)	15 (5.81)	64 (24.42)	258	2
Jiangsu	28 (11.72)	27 (11.30)	23 (9.62)	26 (10.88)	135 (56.49)	239	3
Anhui	5 (4.55)	14 (12.73)	14 (13.64)	5 (4.55)	71 (64.55)	109	4
Fujian	5 (4.67)	17 (15.89)	41 (38.32)	10 (9.35)	34 (31.78)	107	5
Hunan	2 (2.11)	22 (23.16)	2 (2.11)	8 (8.42)	61 (64.21)	95	6
Shanghai	33 (60.00)	8 (14.55)	6 (10.91)	2 (3.64)	6 (10.91)	55	7
Jiangxi	6 (11.32)	2 (3.77)	3 (5.66)	3 (5.66)	39 (73.58)	53	8
Sichuan	0 (0)	0 (0)	0 (0)	0 (0)	39 (100.00)	40	9
Beijing	2 (5.88)	3 (8.82)	2 (5.88)	3 (8.82)	24 (70.59)	34	10
Guangxi	0 (0)	3 (9.38)	0 (0)	0 (0)	29 (90.63)	32	11
Hebei	1 (3.13)	0 (0)	0 (0)	3 (9.38)	28 (87.50)	32	12
Henan	4 (12.50)	0 (0)	0 (0)	1 (3.13)	27 (84.38)	32	13
Hubei	0 (0)	0 (0)	1 (3.13)	1 (3.13)	30 (93.75)	32	14
Shandong	2 (7.14)	3 (10.71)	1 (3.57)	3 (10.71)	19 (67.86)	28	15
Guizhou	0 (0)	1 (5.00)	1 (5.00)	0 (0)	18 (90.00)	20	16
Xinjiang	0 (0)	2 (15.38)	8 (61.54)	0 (0)	3 (23.08)	13	17
Chongqing	0 (0)	0 (0)	0 (0)	0 (0)	9 (100.00)	9	18
Shaanxi	0 (0)	0 (0)	0 (0)	0 (0)	7 (100.00)	7	19
Liaoning	0 (0)	0 (0)	0 (0)	1 (16.67)	5 (83.33)	6	20
Yunnan	0 (0)	0 (0)	0 (0)	0 (0)	6 (100.00)	6	21
Gansu	0 (0)	0 (0)	0 (0)	0 (0)	5 (100.00)	5	22
Tianjin	0 (0)	0 (0)	0 (0)	2 (40.00)	3 (60.00)	5	23
Jilin	0 (0)	2 (66.67)	0 (0)	0 (0)	1 (33.33)	3	24
Shanxi	0 (0)	0 (0)	0 (0)	0 (0)	3 (100.00)	3	25
Xizang	0 (0)	0 (0)	0 (0)	0 (0)	3 (100.00)	3	26
Inner Mongolia	0 (0)	0 (0)	0 (0)	0 (0)	2 (100.00)	2	27
Total	134 (8.63)	302 (19.46)	221 (14.43)	119 (7.67)	776 (50.00)	1553	

### Results of spatial clusters

The global Moran’s *I* indexes of five epidemics were positive and statistically significant. The results identified the spatial distribution of A (H7N9) cases were spatially clustered (Table 3). Moran’s *I* indexes> 0 implied aggregation distribution. Moran’s *I* index of first epidemic was highest (0.610), while the third, fourth and fifth epidemics were higher (around 0.3).

**Table 3:** Global autocorrelation analysis on distribution of avian influenza A (H7N9) in humans across five epidemics in mainland China

Epidemic	Moran’s ***I***	***E(I)***	***Var(I)***	Z Score	***P***-value
1	0.610	−0.034	0.010	6.411	< 0.001
2	0.132	−0.034	0.009	1.742	0.041
3	0.308	−0.034	0.010	3.428	< 0.001
4	0.306	−0.034	0.010	3.454	< 0.001
5	0.336	−0.034	0.012	3.397	< 0.001

Note:*E(I)* was Moran’s *I* expectation, V*ar(I)* was variation of Moran’s *I*

**Fig. 1:**
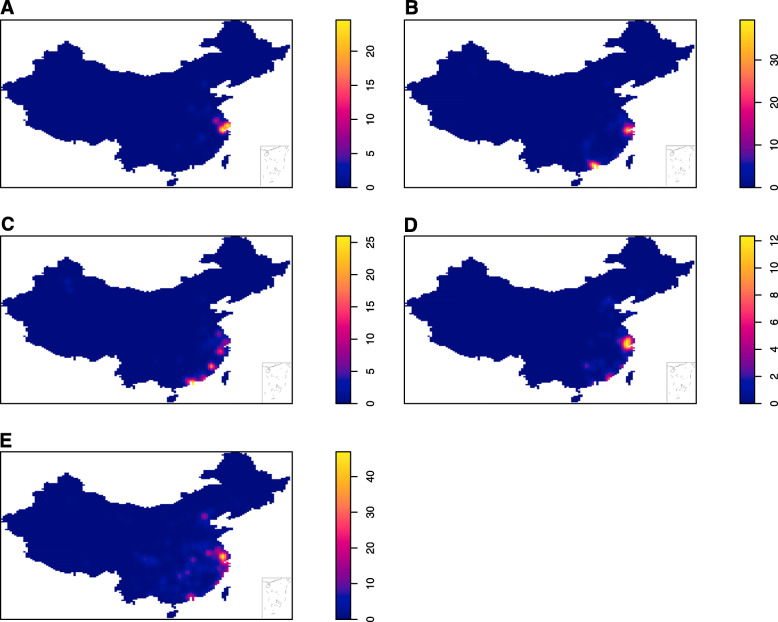
The intensity of A (H7N9) avian influenza in humans across five epidemics. Note: A ~ E showed the intensity of five epidemics. The intensity was represented on a continuous scale of low (blue) to high (yellow). Data sources used in the maps including: confirmed A (H7N9) human cases data was collated from EMPRES-i of FAO (empres-i.fao.org/eipws3g/bioclimatic); the base maps were obtained from the GADM database (https://gadm.org/download_country_v3.html). Maps were built using R software version 3.4.3

Figure 1, maps resulting from the intensity estimate were present for each epidemic, displaying the intensity of the A (H7N9) cases with raster data. The intensity estimates implied that the Yangtze River Delta region and the Pearl River Delta region had the highest intensity, and the intensity shifted gradually across the five epidemic. Initially, the Yangtze River Delta region had higher intensity in the first epidemic; both Yangtze River Delta region and the Pearl River Delta region had higher intensity in the second epidemic; the intensity of Pearl River Delta region was higher than Yangtze River Delta in the third epidemic, whilst small clusters near the regions emerged; the main epidemic were present in the Yangtze River Delta region and the small cluster emerged in inner China in the fourth epidemic; the main cluster was also present in the Yangtze River Delta region and the epidemic region expanded toward north and west in the fifth epidemic.

### Results of temporal clusters

The cases from 2013 to 2017 were combined monthly. The mean angles and standard deviations were calculated and translated into the peak period via the circular distribution method.

Table 4 presented the results of each epidemic for temporal clusters. The peak period was mainly in the winter and spring seasons from the end of December to April. The peak date in first epidemic was in mid-March, and the rest was in February.

**Table 4:** The peak period of avian influenza A (H7N9) in humans in 2013–17

year	Number of cases	*r*	Peak date	Peak period
2013	145	0.840*	19 March	13 February~ 22 April
2014	314	0.779*	6 February	27 December~ 19 March
2015	208	0.743*	11 February	29 December~ 29 March
2016	130	0.554*	26 February	25 December~ 30 April
2017	756	0.700*	27 February	9 January~ 16 April
Total	1553	0.688*	25 February	5 January~ 16 April

“*” showed mean angles were statistically significant, *P* < 0.05

## Discussion

Our study presented the temporal and spatial features of avian influenza A (H7N9) virus in humans across the five epidemic in mainland China through the statistical inferences of the clusters. We demonstrated there were aggregation distributions in the east of China initially, followed by the inner and west of China presented cluster in the fourth and fifth epidemics. Moreover, the peak periods shifted gradually from the first until the fifth epidemic. The results were similar with Dong’s description results [20].

The Yangtze River Delta region and the Pearl River Delta region were higher intensity regions, especially the Yangtze River Delta region. The fourth epidemic has begun to expand to the central China, and the fifth epidemic has expanded to the northern and western regions. There was contest that geographical expansion of A (H7N9) virus implied the human-human epidemic trajectory. However, the evidence was not sufficient. The Yangtze River Delta region is well recognized as the original source for H7N9 outbreaks with the Pearl River Delta region as an additional region across the first three waves [21]. The H7N9 virus may spread silently due to most of them belonging to Low pathogenic avian influenza (LPAI), and then break human-animal interface without disease in poultry [22, 23]. The low pathogenic phenotype in poultry, the strains circulating in local farms and Live poultry markets (LPMs), converted into the high pathogenic phenotype with the four amino acids inserting into the HA cleavage site during the fifth wave [24, 25]. Local density of LPMs was the most important predictor of H7N9 infection risk [22, 26, 27]. LPMs, keeping the avian in crowded conditions which may facilitate avian influenza virus genetic reassortment, have been considered as the reservoir and amplifier for avian influenza viruses [28]. Higher density of markets may exacerbate the risk and explain the strong spatial correlation with H7N9 infection [29]. For the persistent circulation of H7N9 viruses in poultry, poultry trading and movements may contribute to the geographic expansion of the virus [21]. In accordance with the researches, exposure to LPMs was the major epidemic trajectory. Although spatial clusters virtually existed, there has been no evidence of sustained human-to-human transmission so far. To a certain extent, LPM closures were effective in the control of human risk of avian influenza A (H7N9) virus infection [30, 31], which was only a temporary measure for eliminating the source of the infection [30].

The peak incidence was concentrated in February and March, March in first epidemic and February in the following epidemics respectively. The result wasn’t consistent with prior reports. Lei [2] and et al. indicated that the fifth epidemic began earlier and increased rapidly through the description of the epidemics. However, the peak period was not earlier than before via statistical inference in our study. Li [32] and et al. found temperature and rainfall played important roles in the risk of human H7N9 infection, the same as research of Hu [33] and et al. The seasonality of A (H7N9) virus favoring the temperature ranged from approximately 9 °C to 19°Cwas related with the meteorological condition, and the at-risk period may shift gradually across China [33, 34]. For the climate condition, the peak incidence may not shifted significantly.

In response to the epidemic situation, a series of measures and interventions have already been implemented by the national and local authorities. Inter-departmental alliances and effective implementation of evidence-based disease management were crucial to form One Health in China [35]. It is necessary to facilitate the capacity to rapidly detect and contain public health threats at their source via risk communication [36].

Our study had the limitation. Our study analysed only laboratory-confirmed human infections with A (H7N9) virus and excluded the clinically mild cases without confirmed test. Dennis [37] and Yang [38] confirmed there was “clinical iceberg” phenomenon in influenza A (H7N9) in humans through the serological study. Therefore, the full spectrum of human infection may be different from the confirmed cases.

## Conclusions

This analysis provided the statistical inference of spatial and temporal cluster of A (H7N9) epidemic in humans, and presented the aggregation distribution and peak periods. Yangtze River Delta region and the Pearl River Delta region had the spatial cluster and the peak period was from January to April. The spatial scope has begun to expand since the fourth epidemic and temporal heterogeneity varied slightly. With the evaluation, it is necessary to improve the comprehensive collaboration of inter-departmental alliances to monitor continuously, assess risk regularly and communicate epidemic risk.

## Data Availability

The datasets used during the current study are available from the EMPRES Global Animal Disease Information System (EMPRES-i) of FAO (http://empres-i.fao.org/eipws3g/).

## Abbreviations

FAO: Food and Agricultural Organization
LPAI: Low pathogenic avian influenza
LPMs: Live poultry markets
